# A randomized controlled trial of two diets enriched with protein or fat in patients with type 2 diabetes treated with dapagliflozin

**DOI:** 10.1038/s41598-021-90879-z

**Published:** 2021-05-31

**Authors:** Yasuhiro Watanabe, Daisuke Suzuki, Nobuichi Kuribayashi, Daigaku Uchida, Mitsutoshi Kato, Hiroshi Ohashi, Daiji Nagayama, Takashi Yamaguchi, Masahiro Ohira, Atsuhito Saiki, Ichiro Tatsuno

**Affiliations:** 1 Center of Diabetes, Endocrinology and Metabolism, Toho University Sakura Medical Center, 564-1 Shimoshizu, Sakura-shi, Chiba, 285-8741 Japan; 2 Suzuki Diabetes Clinic, 1-3-24 Aikoh, Atsugi-shi, Kanagawa, 243-0035 Japan; 3 Misaki Naika Clinic, 6-44-9 Futawahigashi, Funabashi-shi, Chiba, 274-0805 Japan; 4 Hotaruno Central Naika, 3-30-3 Hotaruno, Kisarazu-shi, Chiba, 292-0038 Japan; 5 Kato Clinic of Internal Medicine, 3-11-14 Takasago, Katsushika-Ku, Tokyo, 125-0054 Japan; 6 Oyama East Clinic, 1-32-1 Ekihigashidori, Oyama-shi, Tochigi, 323-0022 Japan; 7 Nagayama Clinic, 2-12-22 Tenjincho, Oyama-shi, Tochigi, 323-0032 Japan; 8 Chiba Prefectural University of Health Sciences, Wakaba 2-10-1, Mihama-ku, Chiba-shi, 261-0014 Japan

**Keywords:** Diabetes, Randomized controlled trials

## Abstract

Sodium-glucose cotranspsorter-2 (SGLT2) inhibitors (SGLT2i) involve loss of skeletal muscle mass, potentially leading to inadequate HbA1c reduction in type 2 diabetes (T2DM), since muscle mass is related to insulin sensitivity. The benefit of protein-enriched diet for improving HbA1c in SGLT2i-treated T2DM patients remains unclear. We conducted a multicenter, double-blind, randomized, controlled, investigator-initiated clinical trial. 130 T2DM patients treated with dapagliflozin (5 mg) were randomized to isoenergic protein-rich formula diet (P-FD) or fat-rich FD (F-FD) (1:1 allocation) to replace one of three meals/day for 24 weeks. Primary outcome was change in HbA1c. Secondary outcomes were changes in serum insulin, body composition and other metabolic parameters. Although HbA1c decreased significantly in both groups [mean (95% confidence interval) − 0.7% (− 0.9 to − 0.5) in P-FD, − 0.6% (− 0.8 to − 0.5) in F-FD], change in HbA1c was not significantly different between the two groups (P = 0.4474). Fasting insulin and body fat mass decreased, while HDL-cholesterol increased significantly in P-FD, and these changes were significantly greater compared with F-FD (all, P < 0.05). In T2DM treated with dapagliflozin, protein-enriched diet does not contribute to HbA1c reduction, although it decreases serum insulin and body fat mass, and increases HDL-cholesterol compared with fat-enriched diet with identical calories and carbohydrate ratio.

## Introduction

Sodium-glucose cotransporter-2 (SGLT2) inhibitors (SGLT2i) such as dapagliflozin have a glucose-lowering mechanism that does not depend on insulin secretion. The basic pharmacological effect of SGLT2i is promotion of glucose excretion in urine, and the use of SGLT2i corresponds to a decrease of approximately 300 kcal per day due to accelerated urinary excretion of glucose^1^. While administration of SGLT2i increases gluconeogenesis in the liver with loss of glucose from the kidney^2^, fat and muscle are catabolized and ketone bodies are elevated. Thus, the weight loss effect of SGLT2i is thought to involve loss of both fat and lean mass or skeletal muscle in patients with type 2 diabetes mellitus^3–5^. This may lead to an inadequate decrease in HbA1c, because muscle volume correlates with insulin sensitivity^6^.

Whether SGLT2i-induced muscle loss deteriorates insulin sensitivity remains controversial among studies^7–10^. Although excessive supplementation of protein^11^ and fat, especially saturated fatty acids^12^, worsens insulin sensitivity, adequate dietary protein or amino acid supplementation has been reported to prevent loss of muscle mass^13,14^. In addition, some reports have shown that protein-enriched diet improves insulin sensitivity, although few reports have examined the benefit of such diet, especially its contribution to improving HbA1c in type 2 diabetes patients treated with SGLT2i. We investigated whether a protein-enriched diet is beneficial for improving HbA1c in type 2 diabetes patients treated with SGLT2i by comparing with a fat-enriched diet.

## Results

### Baseline characteristics of two groups

**Figure 1:**
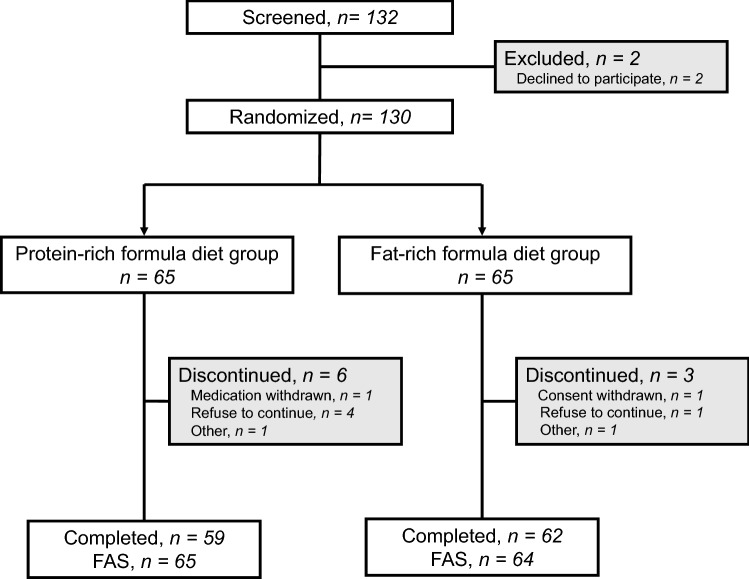
Diagram of participant flow. A total of 121 patients completed the study (59 in protein-rich formula diet group and 62 in fat-rich formula diet group). *FAS* full set analysis.

The participant flow is shown in Fig. 1. Between April 26, 2017 and June 8, 2018, 132 patients were screened, and 130 were enrolled and randomly assigned to a protein-rich formula diet (P-FD) (n = 65) or fat-rich formula diet (F-FD) group (n = 65). The two FDs were started simultaneously with dapagliflozin 5 mg once daily for 24 weeks. There were no significant differences in baseline clinical characteristics between P-FD and F-FD groups (Table 1).

**Table 1:** Baseline clinical characteristics.

	P-FD group	F-FD group	P-value
Number of subjects	65	64	
Male	28 (43.1%)	24 (37.5%)	0.5914
Age (years)	56.9 (11.0)	56.6 (12.0)	0.8831
Body weight (kg)	77.0 (12.9)	76.6 (17.7)	0.8692
BMI (kg/m^2^)	29.4 (4.4)	29.8 (6.3)	0.6897
Past maximum weight (kg)	80.7 (12.8)	81.3 (17.6)	0.8315
Waist circumference (cm)	100.4 (11.3)	99.1 (11.9)	0.5369
Duration of DM (years)	7.5 (6.0)	9.1 (7.9)	0.2317
HbA1c (%)	7.4 (0.4)	7.4 (0.5)	0.8889
HbA1c (mmol/mol)	57.2 (4.6)	57.1 (5.1)	
Fasting glucose (mmol/l)	8.2 (1.7)	8.3 (2.0)	0.8758
eGFR (mL/min/1.73 m^2^)	85.6 (18.9)	85.9 (23.3)	0.9504
sBP (mmHg)	133.2 (16.8)	133.9 (13.7)	0.8133
dBP (mmHg)	80.2 (10.9)	79.1 (8.9)	0.5408
Heart rate (bpm)	77.5 (14.1)	78.0 (14.5)	0.8681
History of hypertension	36 (55.3%)	40 (62.5%)	0.4755
History of dyslipidemia	51 (78.5%)	48 (75.0%)	0.6810

*P-FD* protein-rich formula diet, *F-FD* fat-rich formula diet, *BMI* body mass index, *eGFR* estimated glomerular filtration rate, *sBP* systolic blood pressure, *dBP* diastolic blood pressure. Data are expressed as n (%) or mean (SD).

### Primary outcome measure

**Figure 2:**
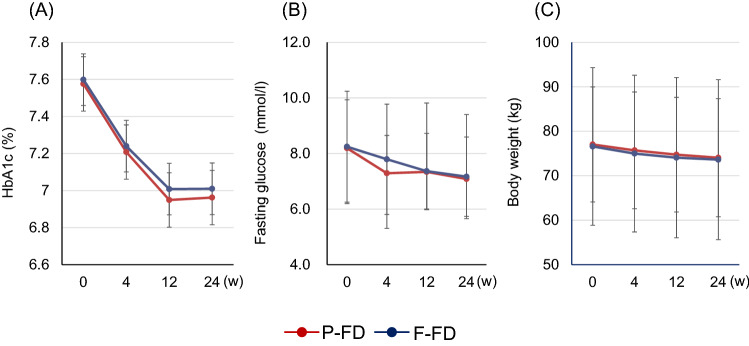
Changes in outcome measures in the group treated with dapagliflozin and protein-rich formula diet (P-FD) and the group treated with dapagliflozin and fat-rich formula diet (F-FD). (**A**) Change in HbA1c, (**B**) change in fasting glucose, (**C**) change in body weight. Data are presented as mean ± SD.

**Figure 3:**
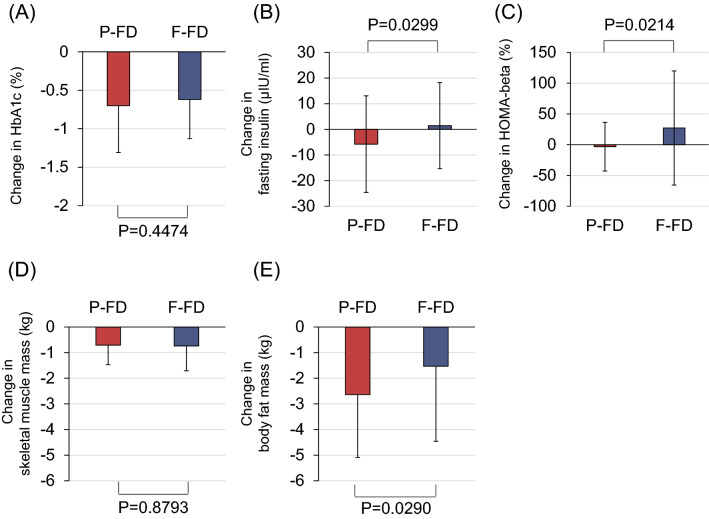
Changes in outcome measures in the group treated with dapagliflozin and protein-rich formula diet (P-FD) and the group treated with dapagliflozin and fat-rich formula diet (F-FD). (**A**) Change in HbA1c, (**B**) change in fasting insulin, (**C**) change in homeostasis model assessment of beta cell function (HOMA-beta), (**D**) change in skeletal muscle mass, (**E**) change in body fat mass. Data are presented as mean ± SD.

Mean HbA1c, fasting glucose, and body weight decreased significantly in a time-dependent manner in both P-FD and F-FD groups (Fig. 2). The primary outcome measure in this study was change in HbA1c. In P-FD group, mean [95% confidence interval (CI)] HbA1c changed from 7.4% (7.3–7.5) at week 0 to 6.7% (6.5–6.8) at week 24 (P < 0.0001); while in F-FD group, mean HbA1c changed from 7.4% (7.3–7.5) at week 0 to 6.8% (6.6–6.9) at week 24 (P < 0.0001). However, the change in HbA1c [week 24–week 0; mean (95% CI)] was not significantly different between P-FD and F-FD groups [− 0.7% (− 0.9 to − 0.5) in P-FD group vs − 0.6% (− 0.8 to − 0.5) in F-FD group, P = 0.4474] (Table 2 and Fig. 3). The dose of dapagliflozin in all patients was 5 mg throughout 24 weeks, and no patient had dose increase to 10 mg.

**Table 2:** Changes in clinical parameters and differences between P-FD group and F-FD group.

	P-FD (Protein: Fat: Carbohydrate = 21:23:56)							F-FD (Protein: Fat: Carbohydrate = 16:29:55)							P-FD vs F-FD
	Week 0		Week 24		Change in value Week 24–Week 0		P	Week 0		Week 24		Change in value Week 24–Week 0		P	P
HbA1c (%)	7.4	(7.3 to 7.5)	6.7	(6.5 to 6.8)	− 0.7	(− 0.9 to − 0.5)	< 0.0001	7.4	(7.3 to 7.5)	6.8	(6.6 to 6.9)	− 0.6	(− 0.8 to − 0.5)	< 0.0001	0.4474
HbA1c (mmol/mol)	57.2	(56.1 to 58.4)	49.5	(47.8 to 51.3)	− 7.6	(− 9.4 to − 5.9)	< 0.0001	57.1	(55.8 to 58.4)	50.5	(48.8 to 52.1)	− 6.8	(− 8.2 to − 5.3)	< 0.0001	0.4474
Fasting glucose (mmol/l)	8.2	(7.7 to 8.6)	7.1	(6.5 to 7.7)	− 1.1	(− 1.8 to − 0.5)	0.0009	8.2	(7.7 to 8.7)	7.2	(6.8 to 7.5)	− 1.1	(− 1.5 to − 0.7)	< 0.0001	0.9614
Body weight (kg)	77.0	(73.8 to 80.2)	74.1	(70.6 to 77.5)	− 3.5	(− 4.3 to − 2.8)	< 0.0001	76.6	(72.2 to 81.0)	73.6	(69.1 to 78.2)	− 2.9	(− 3.6 to − 2.3)	< 0.0001	0.2611
BMI (kg/m^2^)	29.4	(28.3 to 30.45)	28.1	(26.9 to 29.3)	− 1.4	(− 1.7 to − 1.1)	< 0.0001	29.8	(28.2 to 31.4)	28.6	(27.0 to 30.2)	− 1.2	(− 1.4 to − 0.9)	< 0.0001	0.3295
Waist circumference (cm)	100.4	(97.6 to 103.2)	95.7	(92.5 to 98.8)	− 4.4	(− 6.5 to − 2.3)	0.0001	99.1	(96.2 to 102.1)	95.8	(92.4 to 99.2)	− 3.1	(− 4.2 to − 2.0)	< 0.0001	0.2743
sBP (mmHg)	133.2	(129.0 to 137.4)	130.2	(126.8 to 133.5)	− 3.1	(− 7.0 to 0.8)	0.1166	133.9	(130.5 to 137.3)	130.1	(127.0 to 133.2)	− 3.9	(− 7.6 to − 0.3)	0.0348	0.7606
dBP (mmHg)	80.2	(77.5 to 82.9)	79.2	(76.5 to 81.9)	− 1.0	(− 3.3 to 1.3)	0.3727	79.1	(76.9 to 81.4)	79.0	(76.7 to 81.3)	− 0.3	(− 2.8 to 2.2)	0.8194	0.6646
Heart rate (bpm)	77.5	(74.0 to 81.1)	77.6	(74.0 to 81.3)	− 0.4	(− 2.9 to 2.2)	0.7747	78.0	(74.3 to 81.6)	79.7	(75.9 to 83.4)	1.5	(− 1.7 to 4.7)	0.3608	0.3697
Fasting insulin (μIU/ml)	13.0	(8.2 to 17.9)	7.74	(6.53 to 8.95)	− 5.8	(− 10.7 to − 0.8)	0.0232	12.1	(9.0 to 15.2)	13.5	(8.1 to 18.9)	1.5	(− 2.9 to 5.7)	0.5077	0.0299
HOMA-IR	5.3	(2.9 to 7.7)	2.4	(2.0 to 2.8)	− 3.1	(− 5.7 to − 0.6)	0.0176	5.0	(2.7 to 7.3)	4.5	(2.5 to 6.6)	− 0.5	(− 2.8 to 1.8)	0.6720	0.1279
HOMA-beta (%)	54.5	(39.8 to 69.2)	53.0	(42.5 to 63.5)	− 3.23	(− 13.6 to 7.2)	0.5366	55.6	(44.0 to 67.2)	82.7	(52.2 to 113.1)	27.2	(3.4 to 50.9)	0.0257	0.0214
Total cholesterol (mmol/l)	5.0	(4.7 to 5.2)	5.2	(4.9 to 5.4)	0.2	(− 0.0 to 0.3)	0.0765	5.1	(4.9 to 5.4)	5.3	(5.0 to 5.5)	0.1	(− 0.1 to 0.3)	0.2304	0.7917
Triglyceride (mmol/l)	1.6	(1.4 to 1.9)	1.5	(1.2 to 1.7)	− 0.2	(− 0.4 to 0.1)	0.1298	1.8	(1.5 to 2.1)	1.6	(1.4 to 1.8)	− 0.2	(− 0.4 to − 0.0)	0.0297	0.7896
LDL-cholesterol (mmol/l)	3.0	(2.7 to 3.2)	3.0	(2.8 to 3.3)	0.0	(− 0.1 to 0.2)	0.7453	3.1	(3.0 to 3.4)	3.2	(3.0 to 3.4)	0.1	(− 0.1 to 0.2)	0.5048	0.7880
HDL-cholesterol (mmol/l)	1.3	(1.3 to 1.4)	1.5	(1.4 to 1.6)	0.2	(0.1 to 0.3)	< 0.0001	1.3	(1.3 to 1.4)	1.4	(1.3 to 1.5)	0.1	(0.1 to 0.2)	< 0.0001	0.0143
Skeletal muscle mass (kg)	26.8	(25.3 to 28.2)	26.3	(25.0 to 27.7)	− 0.7	(− 0.9 to − 0.5)	< 0.0001	26.5	(24.9 to 28.2)	25.7	(24.1 to 27.4)	− 0.7	(− 1.0 to − 0.5)	< 0.0001	0.8793
Body fat mass (kg)	28.6	(26.7 to 30.6)	26.5	(24.1 to 28.9)	− 2.6	(− 3.3 to − 2.0)	< 0.0001	29.1	(26.2 to 32.1)	27.4	(24.4 to 30.5)	− 1.5	(− 2.3 to − 0.8)	0.0002	0.0290
AST (IU/l)	30.2	(26.0 to 34.5)	24.6	(22.3 to 26.9)	− 5.9	(− 9.1 to − 2.6)	0.0007	27.7	(24.9 to 30.5)	26.4	(23.1 to 29.6)	− 1.5	(− 4.5 to 1.4)	0.3049	0.0513
ALT (IU/l)	40.2	(31.8 to 48.6)	30.4	(25.3 to 35.5)	− 10.9	(− 16.6 to − 5.1)	0.0004	35.8	(31.4 to 40.3)	31.2	(26.6 to 35.9)	− 5.0	(− 9.0 to − 1.1)	0.0138	0.1000
γ-GTP (IU/l)	51.3	(39.5 to 63.1)	39.6	(31.9 to 47.2)	− 13.9	(− 20.8 to − 6.9)	0.0002	53.4	(39.4 to 67.4)	39.3	(29.7 to 48.9)	− 14.3	(− 21.0 to − 7.6)	0.0001	0.9300
Uric acid (mg/dl)	5.3	(4.9 to 5.6)	4.7	(4.4 to 5.0)	− 0.6	(− 0.9 to − 0.4)	< 0.0001	5.3	(5.0 to 5.6)	4.8	(4.5 to 5.1)	− 0.5	(− 0.7 to − 0.3)	< 0.0001	0.4471
BUN (mg/dl)	13.3	(12.4 to 14.2)	16.7	(15.5 to 17.8)	3.3	(2.2 to 4.3)	< 0.0001	13.5	(12.6 to 14.4)	15.0	(14.2 to 15.8)	1.7	(1.0 to 2.4)	< 0.0001	0.0112
Creatinine (mg/dl)	0.66	(0.62 to 0.70)	0.69	(0.64 to 0.73)	0.02	(0.01 to 0.04)	0.0139	0.66	(0.62 to 0.70)	0.68	(0.64 to 0.73)	0.03	(0.01 to 0.04)	0.0002	0.9319
eGFR (mL/min/1.73m^2^)	85.6	(80.9 to 90.3)	82.8	(77.8 to 87.9)	− 3.2	(− 5.8 to − 0.5)	0.0202	85.9	(80.0 to 91.7)	83.1	(76.7 to 89.4)	− 3.3	(− 5.4 to − 1.2)	0.0031	0.9433
Urinary albumin (mg/g Cr)	93.9	(− 5.7 to 193.4)	91.6	(5.5 to 177.7)	− 10.7	(− 50.7 to 29.4)	0.5963	69.3	(39.5 to 99.1)	78.9	(34.0 to 123.8)	− 3.0	(− 37.7 to 31.7)	0.8635	0.7727
Total ketone body (μmol/l)	134.1	(103.84 to 164.4)	209.3	(143.5 to 275.1)	76.5	(10.8 to 142.2)	0.0234	121.8	(87.0 to 156.6)	171.0	(118.1 to 224.0)	48.1	(8.7 to 87.5)	0.0175	0.4602
Acetoacetic acid (μmol/l)	44.0	(34.8 to 53.2)	61.7	(46.1 to 77.4)	19.2	(3.9 to 34.5)	0.0149	39.8	(31.1 to 48.5)	53.5	(40.0 to 67.0)	13.5	(3.8 to 23.3)	0.0075	0.5335
3-hydroxybutyric acid (μmol/l)	90.8	(69.0 to 112.6)	147.6	(96.9 to 198.3)	57.3	(6.2 to 108.5)	0.0287	82.0	(55.7 to 108.3)	117.5	(77.9 to 157.2)	34.5	(4.6 to 64.4)	0.0246	0.4429
hs-CRP (mg/dl)	0.17	(0.13 to 0.21)	0.14	(0.10 to 0.18)	− 0.02	(− 0.06 to 0.01)	0.1675	0.15	(0.12 to 0.18)	0.18	(0.14 to 0.22)	0.03	(− 0.01 to 0.07)	0.1262	0.0391

*P-FD* protein-rich formula diet, *F-FD* fat-rich formula diet, *BMI* body fat mass, *sBP* systolic blood pressure; diastolic blood pressure, *HOMA-IR* homeostasis model assessment of insulin resistance, *LDL* low density lipoprotein, *HDL* high density lipoprotein, *AST* aspartate aminotransferase, *ALT* alanine aminotransferase, *γ-GTP* gamma-glutamyl transpeptidase, *BUN* blood urea nitrogen, *eGFR* estimated glomerular filtration rate, *hs-CRP* high-sensitivity C-reactive protein. Data are expressed in mean (95% CI).

### Secondary outcome measures

The results of secondary endpoints are shown in Table 2. Body weight, body mass index (BMI) and waist circumference decreased significantly after 24 weeks of treatment in both P-FD and F-FD groups, but there were no significant differences in changes [mean (95% CI)] of these parameters between the two groups [change in body weight: − 3.5 kg (− 4.3 to − 2.8) in P-FD vs − 2.9 kg (− 3.6 to − 2.3) in F-FD, P = 0.2611; change in BMI: − 1.4 kg/m^2^ (− 1.7 to − 1.1) in P-FD vs − 1.2 kg/m^2^ (− 1.4 to − 0.9) in F-FD, P = 0.3295; change in waist circumference: − 4.4 cm (− 6.5 to − 2.3) in P-FD vs − 3.1 cm (− 4.2 to − 2.0) in F-FD, P = 0.2743].

Skeletal muscle mass decreased significantly in both groups, but there was no difference in change in skeletal muscle mass between the two groups [− 0.7 kg (− 0.9 to − 0.5) in P-FD vs − 0.7 kg (− 1.0 to − 0.5) in F-FD, P = 0.8793]. Body fat mass decreased significantly in both groups, and the decrease was significantly greater in P-FD group than in F-FD group [− 2.6 kg (− 3.3 to − 2.0) in P-FD vs − 1.5 kg (− 2.3 to − 0.8) in F-FD, P = 0.0290)].

Fasting glucose decreased significantly after 24 weeks of treatment in both P-FD and F-FD groups, but there was no significant difference in change in fasting glucose between the two groups [− 1.1 mmol/l (− 1.8 to − 0.5) in P-FD vs − 1.1 mmol/l (− 1.5 to − 0.7) in F-FD, P = 0.9614]. Fasting insulin decreased significantly after 24 weeks of treatment in P-FD group but not in F-FD group, and a significant difference in change of fasting insulin was observed between the two groups [− 5.8 μIU/ml (− 10.7 to − 0.8) in P-FD vs 1.5 μIU/ml (− 2.9 to 5.7) in F-FD, P = 0.0299]. Homeostatic model assessment (HOMA)-insulin resistance (HOMA-IR) decreased significantly in P-FD group and did not change in F-FD group, although there was no significant difference between the two groups [− 3.1 (− 5.7 to − 0.6) in P-FD group vs − 0.5 (− 2.8 to 1.8) in F-FD group, P = 0.1279]. HOMA-beta cell function (HOMA-beta) increased significantly in F-FD group but did not change in P-FD group, and there was a significant difference between the two groups [27.2% (3.4–50.9) in F-FD group vs − 3.23% (− 13.6 to 7.2) in P-FD group, P = 0.0214]. HDL-cholesterol and blood urea nitrogen (BUN) increased significantly in both groups, and the increases were significantly greater in P-FD group than in F-FD group [HDL-cholesterol: 0.2 mmol/l (0.1–0.3) in P-FD vs 0.1 mmol/l (0.1–0.2) in F-FD, P = 0.0143; BUN: 3.3 mg/dl (2.2–4.3) in P-FD vs 1.7 mg/dl (1.0–2.4) in F-FD, P = 0.0112].

Significant changes in AST, pre-heparin LPL, VLDL, RLP-cholesterol, apo proteins A1 and A2, as well as percent changes in total cholesterol, AST, ALT, RLP-cholesterol, apo proteins A1, A2, C3 and E were observed in P-FD group but not in F-FD group, and these changes were not significantly different between the two groups. Significant changes in systolic blood pressure, triglyceride, and apo protein C2 were observed in F-FD group but not in P-FD group, and these changes were not significantly different between the two groups. Significant increases in levels and percent increases in γ-GTP, uric acid, estimated glomerular filtration rate (eGFR) and blood urea protein, as well as significant increases in creatinine, total ketone body, acetoacetic acid, and 3-hydroxybutyric acid were observed in both P-FD and F-FD groups, but these changes were not significantly different between the two groups.

Although high-sensitivity C-reactive protein (hs-CPR) showed a significant difference between the two groups (P = 0.0391), there was no significant change in hs-CRP in P-FD or F-FD group [− 0.02 mg/dl (− 0.06 to 0.01) in P-FD group (P = 0.1675) vs 0.03 mg/dl (− 0.01 to 0.07) in F-FD group (P = 0.1262)]. The changes in diastolic blood pressure, heart rate, LDL-cholesterol, urinary albumin quantification, apo protein B, ankle branchial index (ABI) and cardio-ankle vascular index (CAVI) were not significantly different between the two groups (see also Supplementary Table S2).

Summarizing the results of secondary outcomes, fasting insulin decreased significantly after 24 weeks in P-FD group. Body fat mass decreased significantly in both groups, while HDL-cholesterol and BUN increased significantly in both groups. The changes in the above four parameters were significantly greater in P-FD group than in F-FD group. On the other hand, HOMA-beta increased in F-FD group but was unchanged in P-FD group, and the change in HOMA-beta was significantly different between the two groups. For parameters other than those described above, there were no significant differences between P-FD and F-FD groups, although there were some significant changes within P-FD group and/or F-FD group.

### Adverse events

There were no severe adverse events in both groups (Supplementary Table S3).

## Discussion

In this study of Japanese type 2 diabetes patients treated with the SGLT2i dapagliflozin, we found that a protein-enriched diet did not contribute to the improvement of HbA1c, although the protein-enriched diet decreased serum insulin and body fat mass, and increased HDL-cholesterol compared with a fat-enriched diet with the same calories and carbohydrate ratio.

The change in HbA1c was not different between P-FD and F-FD groups, but fasting insulin decreased significantly in P-FD and not in F-FD group, with a significant difference between the two groups. This finding may indicate that protein-enriched diet improves insulin sensitivity in type 2 diabetes patients treated with dapagliflozin. Although there are some limitations in using HOMA measures for the evaluation of insulin resistance and beta cell function in patients with diabetes^15^, HOMA-IR can be used reliably for fasting glucose levels lower than 140 mg/dl^16^. The decrease in HOMA-IR was significant only in P-FD group and not in F-FD group, although there was no significant difference between the two groups.

The relation between insulin sensitivity and treatment with SGLT2i is inconsistent among studies. Some studies reported that SGLT2i improved insulin sensitivity, but the degree of improvement varied among type 2 diabetes patients^7–10^. However, other reports showed that SGLT2i did not change insulin sensitivity^17–19^. In addition, the relation between protein-enriched diet and insulin sensitivity in type 2 diabetes remains obscure. Although a report showed that isocaloric diets high in animal or plant protein reduced liver fat and markers of insulin resistance in type 2 diabetes^20^, the results varied depending on the amount, duration, and source of protein^21^. In our study, fasting insulin decreased significantly in P-FD group but did not change in F-FD group with a significant difference between the two groups, indicating that the protein-enriched diet was superior to F-FD diet in improving insulin sensitivity, even though both diets were comparable in the efficiency of lowering HbA1c. Studies have reported that protein-rich supplementation of 25–30% worsens insulin sensitivity^11^, but medium supplementation of 22% improves insulin sensitivity^22^; the latter is very close to the 21% supplementation in our P-FD group. These findings suggest the existence of an optimal dose of protein supplementation and/or protein/fat ratio for the improvement of insulin sensitivity. The increase in insulin sensitivity by protein supplementation may be linked to the increase in HDL-cholesterol as observed in this study, because the metabolism of HDL-cholesterol is associated with insulin sensitivity^23^.

Since the proteins used in FD are derived from casein, whey protein and soybean, it is possible that amino acid loading via protein supplementation may decrease serum insulin. As a mechanism by which amino acids improve insulin sensitivity, supplementation of amino acids, particularly branched chain amino acids, may suppress muscle loss^13,14^. Supplementation of amino acids not only suppresses muscle loss but also improves insulin sensitivity^22,24^, although we observed no significant difference in the decrease of skeletal muscle mass between P-FD and F-FD groups. Overloading of fat, especially saturated fatty acids, in meal has been reported to worsen insulin sensitivity and increase HbA1c^12^. The P/F/C ratio of total daily diet in F-FD group is close to that of Japanese standard diet for the treatment of type 2 diabetes^25^. The source of fat used in this study is coconut oil which is reported to improve insulin sensitivity^26^. Therefore, fat supplementation appears to be neither excessive in amount nor poor in quality. It is possible that protein-enriched diet rather than fat-enriched diet contributed to the decrease of serum insulin in P-FD group, and that changing the protein/fat ratio per se may be beneficial.

Another finding is the significantly greater reduction in body fat mass in P-FD group compared to F-FD group. For the evaluation of body composition, we used the bioelectrical impedance analysis (BIA) method instead of the dual energy X-ray absorptiometry (DXA) method, considering the easy availability of the BIA method to general practitioners. BIA method has been reported to correlate well with the DXA method^27,28^ (see also Supplementary Appendix 4). Dietary protein intake has been shown to be beneficial for reduction of body fat mass^29^, and we previously reported that protein-enriched FD decreased visceral fat area and serum insulin accompanied by HbA1c reduction in type 2 diabetes patients^30^. Whole body energy expenditure, increment of fat oxidation, thermogenic effect by promoted satiety are assumed to be the mechanisms by which protein intake reduces body fat^31–33^.

Although similar reduction in HbA1c was observed in both groups under treatment with dapagliflozin, a decrease in fasting insulin was observed in P-FD group and not in F-FD group, with a significant difference between the two groups. This suggests that F-FD group required more insulin secretion for the same reduction in HbA1c compared with P-FD group, which was also supported by the increase in HOMA-beta. High blood glucose impairs insulin secretion (glucose toxicity), and SGLT2 inhibitors lower blood glucose through promoting glucosuria to increase insulin secretion^34,35^. The F-FD group required more insulin secretion to lower HbA1c to the same degree as in the P-FD group. This may not be a beneficial effect for long-term blood glucose control.

There are some limitations in this study. First, although this study focuses on changes in metabolism and body composition when changing the protein to lipid ratio in FD given to patients using SGLT2 inhibitors, the additive effects of P-FD when given concurrently with dapagliflozin should be evaluated by comparing with dapagliflozin alone and not with another FD. Comparison between three groups was difficult in the present clinical trial given the limited number of patients and limited resources, and this will be our future task. Second, the subjects of this study were all Japanese patients and the results cannot be generalized universally. Third, since the type 2 diabetes patients in this study had a relatively short duration of diabetes, were in the 50 s, and had an obese tendency for Asians, they might have preserved insulin secretion capacity, which would have made it difficult to demonstrate a significant difference in the primary endpoint between the two groups. Fourth, the study period was only 24 weeks and the difference in protein/fat ratio between the group was small. Different results may be obtained if the subjects are observed for a prolonged period or if FDs with greater differences in protein/fat ratio are used. Fifth, the subjects took P-FD or F-FD once a day, while the calorie intake of the other two meals was managed by the subjects with instructions from a nutritionist or nurse. There may be some discrepancy between the instructed calorie and the actually ingested calorie.

In conclusion, in type 2 diabetes patients treated with dapagliflozin, an isoenergic protein-enriched diet does not contribute to the change in HbA1c, although it decreases serum insulin and body fat mass, and increases HDL-cholesterol compared with a fat-enriched diet with the same calories and carbohydrate ratio.

## Methods

### Study design

The study design was a multicenter, double-blind, randomized, controlled, investigator-initiated clinical trial. This study was registered as “Diet-Dapper Study” in the University Hospital Medical Information Network (UMIN) Clinical Trials Registry (UMIN000024580) on 1/3/2017. Seven facilities participated in this study (all facilities are listed in Supplementary Information). This study was conducted in compliance with the principles of the Declaration of Helsinki. Institutional review board (IRB) approval was obtained from the Ethics Committee of Toho University Sakura Medical Center (ID number: S16101) on 16/3/2017, the Ethics Committee of Shin-Oyama City Hospital, and the centralized IRB for the other facilities. The protocol was reported previously^36^. To conduct a double-blind, randomized, controlled trial comparing diets with identical calories and carbohydrate ratio, we used FD. Formula diet contains low carbohydrate, low fat, and sufficient protein, vitamins and minerals to support a healthy and balanced diet, and was originally developed for treating severe obesity as a low-calorie food^37–40^. Dapagliflozin was used as the SGLT2i.

Subjects were randomized to receive a protein-rich FD (P-FD) or a newly developed fat-rich FD (F-FD) to replace one of three meals/day for 24 weeks. The two FDs had the same calories (182 kcal). Subjects started the FD and dapagliflozin simultaneously. In P-FD group, the patients took 5 mg of dapagliflozin orally once daily (the initial dose for the treatment of type 2 diabetes in Japan), and they replaced one meal with P-FD while taking two standard meals a day. In F-FD group, patients took the same dose of dapagliflozin and replaced one meal per day with F-FD. There was no restriction on when the patients took the FD (breakfast, lunch, or dinner) in both groups. In principle, a daily dose of 5 mg of dapagliflozin was continued, but if HbA1c exceeded 8.5%, the dose would be increased to 10 mg at the investigator’s discretion. The study period for both groups was 24 weeks. Written informed consent was obtained from all participants. Eligible patients were randomized to P-FD or F-FD group at a ratio of 1:1 by a computer program installed at the registration center. Randomization used a minimization method balancing age (≤ 65 or > 65 years), HbA1c level (≤ 8.0 or > 8.0%) and waist circumference (men: ≤ 85 or > 85 cm; women: ≤ 80 or > 80 cm) at the time of screening.

### Formula diets and calorie intake

The P-FD and F-FD were purchased from Sunny Health Co., Ltd. The FD was reconstituted in a 600-ml dedicated shaker. A package of the FD was added to 350–400 ml of water and shaken well before consumption. No significant difference in taste between P-FD and F-FD was confirmed by Sunny Health Co., Ltd. before the start of the trial. On weeks 0 and 12, the subjects received instructions on how to use the FD and nutritional guidance about calorie intake from a nutritionist at each facility. In the absence of a nutritionist, the instructions were given by nurses.

To calculate the standard calorie intake for the two conventional meals a day, we used the standard daily calorie intake of 35 kcal/kg × standard body weight (kg), assuming that the standard body weight was equivalent to BMI of 22 kg/m^2^. Essentially, the diet for type 2 diabetes patients was calculated at 25–30 kcal per standard body weight. However, due to the low calorie content of FD, we set the non-FD meals at 35 kcal so that the total daily calorie would not be too low. For example, if a patient's standard body weight was 60 kg, standard daily calorie intake would be 2100 kcal per day or 700 kcal per meal at 35 kcal per standard body weight. Therefore, if the subject took FD (182 kcal) once a day and a normal meal of 700 kcal twice a day, the total calorie was 1582 kcal (182 kcal + 700 kcal + 700 kcal) per day, or 26.4 kcal/kg. The protein components of FD consisted of casein, whey protein, and soybean protein, and the fat components consisted of coconut oil containing both polyunsaturated fatty acids and saturated fatty acids. The P-FD contained 19.4 g of protein, 2.4 g of fat, and 24.2 g of carbohydrate. The F-FD contained 1.1 g of protein, 11.0 g of fat, and 23.6 g of carbohydrate. The detailed components of P-FD and F-FD are shown in Supplementary Table S1. The protein/fat/carbohydrate (P/F/C) ratio of total daily diet consumed by the subjects was 21:23:56 (protein 1.4 g/kg and fat 0.7 g/kg) in P-FD group and 16:29:55 (protein 1.1 g/kg and fat 0.8 g/kg) in F-FD group. The investigators confirmed the status of compliance to medication and FD intake at each visit and instructed the subjects to bring empty packages of the FD and unused FD at the next visit. In this study, participants were not given intervention related to exercise therapy. Those who had a regular exercise routine were allowed to continue, and those who did not have a regular exercise routine were not instructed to start a new exercise regimen.

### Eligibility criteria

Eligible patients were type 2 diabetes patients who satisfied all the following inclusion criteria: (1) aged between 20 and 75 years when providing consent; (2) HbA1c in the range of 7.0–8.5%; (3) BMI > 22 kg/m^2^, (4) estimated glomerular filtration rate eGFR ≥ 45 ml/min/1.73 m^2^; (5) had adequate understanding of the study contents upon receiving detailed explanations based on the written consent form, and gave written informed consent of their own free will. The detail exclusion criteria are provided in Supplementary Appendix 1. In principle, treatments for diabetes, hypertension, dyslipidemia, and hyperuricemia were not changed from the time of obtaining consent to the completion of study, to avoid effects on the efficacy and safety assessments of this study.

### Outcome measures

The primary outcome measure was the change in HbA1c from the start of treatment (week 0) to 24 weeks after initiation of treatment. Secondary outcome measures included changes in body weight, BMI, waist circumference, fasting glucose, fasting insulin, HOMA-beta and HOMA-IR, serum lipid level, ketone fraction, hs-CRP, urinary albumin quantification, and body composition (BIA). The details of secondary outcomes are given in Supplementary Appendices 2, 3 and 4.

### Sample size and statistical analysis

The primary endpoint of this study was the change in HbA1c. Shirai et al.^30^ reported a change in HbA1c of − 0.6 ± 1.1% using P-FD. According to a Japanese phase III clinical trial of dapagliflozin^41^, the change in HbA1c after 24-week treatment with dapagliflozin was − 0.4% ± 0.7%. There is no report comparing the effects of protein-rich FD and fat-rich FD in improving HbA1c. In this study, we hypothesized that P-FD used with dapagliflozin would have additive HbAlc lowering effect compared with dapagliflozin alone. Referring to the 0.4% HbAlc reduction by dapagliflozin in the Japanese phase III study^41^ and 0.6% HbA1c reduction by P-FD reported by Shirai et al.^30^, we estimated that 1.0% reduction in HbAlc would be achieved in P-FD group. In F-FD group, HbA1c reduction was predicted to be 0.6%, which was the sum of 0.4% HbA1c reduction by dapagliflozin^41^ and 0.2% HbA1c reduction by conventional Japanese diet reported by Shirai et al.^30^. The standard deviation of the change in HbA1c is presumed to be 0.8%, which is the range reported in clinical studies^34,42^. At a significance level (a) of 0.05 and a detection power [100(1 − b)] of 80%, the number of subjects per group necessary to detect a significant difference in change in HbA1c between the two groups was calculated to be 64. Assuming 5% deviant and omission samples, the target number of subjects for each group was 68, with a total of 138 participants in this study.

Summary statistics of the background data were calculated for each group. Patient characteristics were compared using χ^2^ test or Fisher’s exact test for categorical variables, and Student’s t test or Wilcoxon rank sum test for continuous variables. In the analyses of primary and secondary outcomes, summary statistics (number of subjects, mean, standard deviation, minimum, median, and maximum) were calculated for the measured value and the amount of change for each evaluation item. The amount of change was compared using one-sample t test for intragroup comparison and two-sample t test for intergroup comparison. All the analyses were pre-specified as a part of the protocol^36^.

## Supplementary Information

Supplementary Information.
