# Supplementary material for: A randomized controlled trial of two diets enriched with protein or fat in patients with type 2 diabetes treated with dapagliflozin
Source: Sci Rep. 2021 May 31;11:11350. doi: 10.1038/s41598-021-90879-z (PMC8166978; doi:10.1038/s41598-021-90879-z)
Supplement: Supplementary file 1 — Supplementary Information. [file 41598_2021_90879_MOESM1_ESM.docx]

**Online Supporting Material**

**A randomized controlled trial of two diets enriched with protein or fat in patients with type 2 diabetes treated with dapagliflozin**

Yasuhiro Watanabe, Daisuke Suzuki, Nobuichi Kuribayashi, Daigaku Uchida, Mitsutoshi Kato, Hiroshi Ohashi, Daiji Nagayama, Takashi Yamaguchi, Masahiro Ohira, Atsuhito Saiki, Ichiro Tatsuno*

Yasuhiro Watanabe, MD, PhD

Takashi Yamaguchi, MD, PhD

Masahiro Ohira, MD, PhD

Atsuhito Saiki, MD, PhD

Ichiro Tatsuno, MD, PhD*

Center of Diabetes, Endocrinology and Metabolism, Toho University Sakura Medical Center, Chiba, Japan

564-1 Shimoshizu, Sakura-shi, Chiba 285-8741, Japan

Daisuke Suzuki, MD

Suzuki Diabetes Clinic, Kanagawa, Japan

1-3-24 Aikoh, Atsugi-Shi, Kanagawa 243-0035, Japan

Nobuichi Kuribayashi, MD

Misaki Naika Clinic, Chiba, Japan

6-44-9 Futawahigashi, Funabashi-shi, Chiba 274-0805, Japan

Daigaku Uchida, MD

Hotaruno Central Naika, Chiba, Japan

3-30-3 Hotaruno, Kisarazu-shi, Chiba 292-0038, Japan

Mitsutoshi Kato, MD

Kato Clinic of Internal Medicine, Tokyo, Japan

3-11-14 Takasago, Katsushika-Ku, Tokyo 125-0054, Japan

Hiroshi Ohashi, MD

Oyama East Clinic, Tochigi, Japan

1-32-1 Ekihigashidori, Oyama-shi, Tochigi 323-0022, Japan

Daiji Nagayama, MD, PhD

Nagayama Clinic, Tochigi, Japan

2-12-22 Tenjincho, Oyama-shi, Tochigi 323-0032, Japan

Corresponding author:

Ichiro Tatsuno, MD, PhD

Center of Diabetes, Endocrinology and Metabolism, Toho University Sakura Medical Center, 564-1 Shimoshizu, Sakura-shi, Chiba 285-8741, Japan

Tel: +81-43-462-8811, Fax: +81-43-462-8820

Chiba Prefectural University of Health Sciences, Wakaba 2-10-1, Mihama-ku, Chiba-shi, 261-0014, Japan

TEL: +81-43-296-2000, Fax: +81-43-272-1716

E-mail: ichiro.tatsuno@med.toho-u.ac.jp

E-mail: ichirotatsuno@gmail.com

**Appendix 1**

**Exclusion criteria**

Patients who met any of the following exclusion criteria were excluded from this study: (1) type 1 diabetes; (2) use of sodium-glucose cotransporter-2 inhibitors within the previous 3 months; (3) hypersensitivity to the drug components; (4) severe ketosis, diabetic coma or pre-coma; (5) treated with insulin; (6) determined by the investigator as inappropriate to participate in the study; (7) eGFR less than 45 ml/min/1.73 m^2^; (8) planning to become pregnant; (9) pregnant, possibly pregnant, or breast-feeding; (10) severe infectious disease, severe trauma, preoperative or postoperative patient; (11) allergy to the ingredients of the formula diets (soybeans, eggs, yeasts and milk).

**Appendix 2**

**Secondary outcomes**

(1) body weight and BMI; (2) blood pressure and heart rate; (3) waist circumference; (4) fasting blood glucose level and insulin level for calculation of homeostatic model assessment (HOMA)-beta cell function (HOMA-β) and HOMA-insulin resistance (HOMA-IR); (5) serum lipid levels (total cholesterol, LDL-cholesterol calculated using the Friedewald equation, fasting triglyceride, HDL-cholesterol); (6) ketone fraction (venous blood); (7) lipid-related parameters [pre-heparin lipoprotein lipase (LPL) protein, lipoprotein fraction (polyacrylamide gel electrophoresis), remnant-like lipoprotein cholesterol, apoprotein (Apo A1, A2, B, C2, C3, E)]; (8) high sensitivity C-reactive protein (hs-CRP); (9) urinary albumin quantification; (10) ankle brachial index (ABI) and cardio ankle vascular index (CAVI) as a marker of arterial stiffness (only when measurement is possible), (11) body composition (only when measurement is possible). Insulin, ketone fraction, lipid-related parameters, hs-CRP were measured at a central laboratory (LSI Medience Corporation, Tokyo, Japan). ABI and CAVI were measured using the VaSera series (FUKUDA DENSHI, Tokyo, Japan). Body composition was evaluated by the InBody series (Biospace, Tokyo, Japan) that performs multifrequency bioelectrical impedance analysis. Serious adverse events (AEs) were documented and reported per regulatory requirements.

**Appendix 3**

**Cardio ankle vascular index** (**CAVI)**

A novel arterial stiffness diagnostic parameter called CAVI has been developed in Japan, which essentially reflects the stiffness of the aorta, femoral artery, and tibial artery^1^. This stiffness parameter has been reported to be less affected by blood pressure at the time of measurement and adequately reproducible for clinical use^2^. CAVI is also known to be associated with traditional parameters of vascular structure and function, such as intima-media thickness (IMT), pulse wave velocity (PWV), and central augmentation index^3^. Furthermore, no special technique is required for the measurement of CAVI. CAVI has been associated with a number of risk factors for cardiovascular disease (CVD) and the severity of CVD^4-8^. An increase in number of risk factors for metabolic syndrome also correlates positively with increased CAVI, probably due to insulin resistance^9^.

**Appendix 4**

**Body composition measurement**

Body fat is measured by bioelectrical impedance analysis (BIA) using InBody series (Biospace, Tokyo, Japan). The BIA method is noninvasive and easy to measure and is more convenient and effective when measuring multiple times. The BIA method measures the resistance (impedance) of the body by applying a weak and noninvasive current to the body. Since the muscle contains water that readily conducts electricity, the impedance decreases as the amount of muscle increases, and conversely the impedance increases as fat increases. Impedance in the body is measured and the impedance index is calculated by an established formula^10^. Body water content is calculated from this impedance index, and lean body mass is determined based on the fact that approximately 73% of the fat-free mass is body water. Further, the body fat mass is obtained by subtracting the fat-free mass from the body weight.

**Supplementary Table S1: The detailed composition of protein-rich formula diet (P-FD) and fat-rich formula diet (F-FD)**

|  |  | Nutritional components | |
| --- | --- | --- | --- |
|  | unit | P-FD | F-FD |
| Capacity | g | 50 | 39 |
| Energy | kcal | 182 | 182 |
| Protein | g | 19.4 | 1.1 |
| Fat | g | 2.4 | 11 |
| Carbohydrate | g | 24.2 | 23.6 |
| Sugar | g | 17.3 | 15.6 |
| Dietary fiber | g | 7 | 8 |
| Sodium | mg | 264 | 145 |
| Phosphorus | mg | 159 | 34 |
| Iron | mg | 6 | 5 |
| Calcium | mg | 371 | 230 |
| Potassium | mg | 820 | 760 |
| Magnesium | mg | 113 | 110 |
| Copper | mg | 0.59 | 0.45 |
| Zinc | mg | 5.9 | 5.3 |
| Iodine | μg | 60 | 55 |
| Vitamin A | μg | 438 | 406 |
| Vitamin B1 | mg | 0.93 | 0.96 |
| Vitamin B2 | mg | 1.11 | 1.06 |
| Vitamin B6 | mg | 1.12 | 1.22 |
| Vitamin B12 | μg | 2.9 | 2.5 |
| Vitamin C | mg | 65 | 59 |
| Vitamin D | μg | 4.3 | 4.2 |
| Vitamin E | mg | 6.4 | 6.9 |
| Vitamin K | μg | 126 | 96 |
| Folic acid | μg | 195 | 148 |
| Pantothenic acid | mg | 3.33 | 3.12 |
| Niacin | mg | 11.9 | 7.7 |

**Supplementary Table S2: Changes in clinical parameters and differences between P-FD and F-FD groups**

|  | **P-FD**  **(Protein: Fat: Carbohydrate = 21:23:56)** | | | | | | | **F-FD**  **(Protein: Fat: Carbohydrate = 16:29:55)** | | | | | | | **P-FD**  **vs**  **F-FD** |
| --- | --- | --- | --- | --- | --- | --- | --- | --- | --- | --- | --- | --- | --- | --- | --- |
|  | Week 0 | | Week 24 | | Change in value Week 24 ‒ Week 0 | | P | Week 0 | | Week 24 | | Change in value Week 24 ‒ Week 0 | | P | P |
| pre-heparin LPL (ng/ml) | 57.9 | (52.7 to 63.0) | 66.2 | (60.1 to 72.3) | 8.4 | (4.3 to 12.6) | 0.0001 | 58.9 | (54.1 to 63.7) | 61.9 | (57.0 to 66.7) | 3.4 | (-0.3 to 7.0) | 0.0710 | 0.0693 |
| polyacrylamide gel electrophoresis |  |  |  |  |  |  |  |  |  |  |  |  |  |  |  |
| HDL (%) | 24.1 | (22.6 to 25.5) | 26.6 | (24.7 to 28.4) | 2.6 | (1.6 to 3.5) | <0.0001 | 22.7 | (21.5 to 23.9) | 24.3 | (22.8 to 25.8) | 1.7 | (0.6 to 2.9) | 0.0039 | 0.2720 |
| LDL (%) | 49.4 | (47.4 to 51.5) | 49.7 | (47.8 to 51.5) | -0.4 | (-2.2 to 1.4) | 0.6720 | 49.5 | (47.7 to 51.3) | 49.2 | (47.4 to 51.0) | -0.5 | (-2.0 to 1.0) | 0.5032 | 0.9224 |
| IDL (%) | 14.3 | (12.6 to 16.0) | 13.6 | (12.2 to 15.1) | -0.6 | (-2.2 to 0.9) | 0.4168 | 13.7 | (12.5 to 14.9) | 14.7 | (13.5 to 15.9) | 0.7 | (-0.7 to 2.0) | 0.3394 | 0.2130 |
| VLDL (%) | 16.0 | (14.6 to 17.3) | 14.2 | (12.8 to 15.5) | -1.8 | (-3.1 to -0.5) | 0.0069 | 16.1 | (14.9 to 17.3) | 14.9 | (13.6 to 16.2) | -0.9 | (-2.0 to 0.3) | 0.1296 | 0.2937 |
| RLP-cholesterol (mg/dl) | 7.7 | (6.1 to 9.2) | 5.8 | (4.8 to 6.8) | -1.8 | (-2.9 to -0.6) | 0.0027 | 8.2 | (6.69 to 9.72) | 7.0 | (5.81 to 8.11) | -1.0 | (-2.0 to 0.0) | 0.0534 | 0.3105 |
| Apo protein A1 (mg/dl) | 137.7 | (133.1 to 142.1) | 141.8 | (136.2 to 147.2) | 4.6 | (1.6 to 7.5) | 0.0029 | 136.8 | (132.5 to 141.0) | 137.0 | (132.7 to 141.3) | 1.0 | (-2.0 to 4.0) | 0.5016 | 0.0883 |
| Apo protein A2 (mg/dl) | 31.1 | (29.9 to 32.2) | 32.9 | (31.3 to 34.5) | 1.8 | (0.7 to 2.9) | 0.0014 | 31.1 | (30.1 to 32.1) | 31.6 | (30.4 to 32.8) | 0.5 | (-0.3 to 1.4) | 0.1914 | 0.0650 |
| Apo protein B (mg/dl) | 88.0 | (82.3 to 93.8) | 89.2 | (83.5 to 94.8) | 0.5 | (-3.0 to 3.9) | 0.7806 | 92.2 | (87.0 to 97.4) | 94.6 | (89.4 to 99.8) | 2.0 | (-2.1 to 6.1) | 0.3315 | 0.5692 |
| Apo protein C2 (mg/dl) | 5.5 | (5.0 to 6.1) | 5.9 | (5.3 to 6.4) | 0.3 | (-0.1 to 0.7) | 0.1135 | 5.9 | (5.4 to 6.5) | 6.45 | (5.9 to 7.0) | 0.6 | (0.2 to 1.0) | 0.0040 | 0.3002 |
| Apo protein C3 (mg/dl) | 11.1 | (10.2 to 11.9) | 11.7 | (10.7 to 12.7) | 0.6 | (-0.2 to 1.4) | 0.1498 | 12.1 | (11.0 to 13.1) | 12.2 | (11.3 to 13.2) | 0.4 | (-0.4 to 1.2) | 0.3379 | 0.7390 |
| Apo protein E (mg/dl) | 3.6 | (3.3 to 4.0) | 3.2 | (3.0 to 3.4) | -0.4 | (-0.6 to -0.1) | 0.0009 | 3.7 | (3.4 to 4.0) | 3.4 | (3.1 to 3.6) | -0.2 | (-0.4 to -0.1) | 0.0083 | 0.3166 |
| CAVI | 8.4 | (8.0 to 8.9) | 8.4 | (7.9 to 8.8) | -0.1 | (-0.4to 0.2) | 0.5353 | 8.3 | (7.9 to 8.7) | 8.2 | (7.8 to 8.6) | -0.1 | (-0.3 to 0.1) | 0.3733 | 0.9140 |
| ABI | 1.1 | (1.1 to 1.1) | 1.1 | (1.1 to 1.1) | 0.0 | (-0.0 to 0.02) | 0.8400 | 1.1 | (1.1 to 1.1) | 1.1 | (1.1 to 1.1) | -0.0 | (-0.0 to 0.0) | 0.3465 | 0.4114 |

P-FD, protein-rich formula diet; F-FD, fat-rich formula diet; LPL, lipoprotein lipase; HDL, high-density lipoprotein; LDL, low-density lipoprotein; IDL; intermediate-density lipoprotein; VLDL, very low-density lipoprotein; RLP, remnant-like particles; CAVI, cardio-ankle vascular index; ABI, ankle-brachial pressure index, wk, week. Data are presented as mean (95% CI).

**Supplementary Table S3: Occurrence of adverse events**

|  | | **P-FD group** | | **F-FD group** | |
| --- | --- | --- | --- | --- | --- |
|  |  | **No. of cases** | **(%)** | **No. of cases** | **(%)** |
| Target cases | | 65 |  | 64 |  |
| Adverse event (no. of cases) | | 6 | (9.2) | 3 | (4.7) |
| Number of adverse events | | 11 | (16.9) | 3 | (4.7) |
|  | low blood pressure | 1 | (1.5) | 0 | (0) |
|  | diarrhea | 1 | (1.5) | 0 | (0) |
|  | hypoglycemic symptoms | 1 | (1.5) | 0 | (0) |
|  | cystitis | 2 | (3.1) | 2 | (3.1) |
|  | frequent urination | 1 | (1.5) | 0 | (0) |
|  | penis sore | 1 | (1.5) | 0 | (0) |
|  | itching of vulva | 1 | (1.5) | 0 | (0) |
|  | bullous pemphigoid | 2 | (3.1) | 0 | (0) |
|  | vulvovaginal candidiasis | 1 | (1.5) | 0 | (0) |
|  | foot fracture | 0 | (0) | 1 | (1.6) |

P-FD, protein-rich formula diet; F-FD, fat-rich formula diet

**References**

1. Shirai K, Utino J, Otsuka K, Takata M: A novel blood pressure-independent arterial wall stiffness parameter; cardio-ankle vascular index (CAVI). J Atheroscler Thromb 13: 101-107, 2006

2. Matsui Y, Kario K, Ishikawa J, Eguchi K, Hoshide S, Shimada K: Reproducibility of arterial stiffness indices (pulse wave velocity and augmentation index) simultaneously assessed by automated pulse wave analysis and their associated risk factors in essential hypertensive patients. Hypertens Res 27: 851-857, 2004

3. Gomez-Sanchez L, Garcia-Ortiz L, Patino-Alonso MC, et al.: The Association Between the Cardio-ankle Vascular Index and Other Parameters of Vascular Structure and Function in Caucasian Adults: MARK Study. J Atheroscler Thromb 22: 901-911, 2015

4. Kubozono T, Miyata M, Ueyama K, et al.: Clinical significance and reproducibility of new arterial distensibility index. Circ J 71: 89-94, 2007

5. Nakamura K, Tomaru T, Yamamura S, Miyashita Y, Shirai K, Noike H: Cardio-ankle vascular index is a candidate predictor of coronary atherosclerosis. Circ J 72: 598-604, 2008

6. Noike H, Nakamura K, Sugiyama Y, et al.: Changes in cardio-ankle vascular index in smoking cessation. J Atheroscler Thromb 17: 517-525, 2010

7. Suzuki J, Kurosu T, Kon T, Tomaru T: Impact of cardiovascular risk factors on progression of arteriosclerosis in younger patients: evaluation by carotid duplex ultrasonography and cardio-ankle vascular index(CAVI). J Atheroscler Thromb 21: 554-562, 2014

8. Dobsak P, Soska V, Sochor O, et al.: Increased cardio-ankle vascular index in hyperlipidemic patients without diabetes or hypertension. J Atheroscler Thromb 22: 272-283, 2015

9. Satoh N, Shimatsu A, Kato Y, et al.: Evaluation of the cardio-ankle vascular index, a new indicator of arterial stiffness independent of blood pressure, in obesity and metabolic syndrome. Hypertens Res 31: 1921-1930, 2008

10. Malbrain ML, Huygh J, Dabrowski W, De Waele JJ, Staelens A, Wauters J: The use of bio-electrical impedance analysis (BIA) to guide fluid management, resuscitation and deresuscitation in critically ill patients: a bench-to-bedside review. Anaesthesiol Intensive Ther 46: 381-391, 2014
